# Intravenous patient-controlled analgesia regimen in postoperative pain management following elective cesarean section: A single-center retrospective evaluation

**DOI:** 10.1097/MD.0000000000033474

**Published:** 2023-04-14

**Authors:** Mi Roung Jun, Moon Ok Lee, Haeng Seon Shim, Jeong Won Park, Jeong Yeon Kim, Sungbo Shim, Jihoon Lee

**Affiliations:** a Department of Anesthesiology and Pain Medicine, Gyeongsang National University Hospital, Gyeongsang National University College of Medicine, Jinju, Korea; b Department of Anesthesiology and Pain Medicine, Samsung Changwon Hospital, Sungkyunkwan University School of Medicine, Changwon, Korea.

**Keywords:** analgesia, cesarean section, fentanyl, patient-controlled

## Abstract

Intravenous patient-controlled analgesia (IV PCA; IVA) is the most widely used method for postoperative pain management. An appropriate IVA regimen is required, depending on the expected intensity of pain after surgery. This study expected that a decrease in the second prescription rate of IVA after elective cesarean section (CS) would help establish an appropriate regimen for the initial IVA. We retrospectively reviewed the records of 632 patients who were prescribed IVA after CS. We classified patients into phase 1 (basal rate 15.00 mcg/hours, bolus dose 15.00 mcg, total volume 100 mL) and phase 2 (basal rate 31.25 mcg/hours, bolus dose 31.25 mcg, nefopam 60 mg, paracetamol 3 g, total volume 160 mL) according to the IVA regimen, and patients in phase 2 were classified into the basal 15 group and basal 30 group according to the basal rate of IVA. We compared the rates of second prescription, drug removal, and side effects of IVA between the 2 phases and the 1 group. We analyzed the data of 631 eligible patients. The second prescription rate of IVA in phase 2 was 3.77%, a significant decrease compared to that in phase 1 (27.48%); however, the incidence of complications in phase 2 was 6.92%, a significant increase compared to that in phase 1 (0.96%). Within phase 2, in the basal 30 group, the basal rate was almost double that in the basal 15 group. However, there were no significant differences in the rate of second prescription, removed drug IVA, or adverse events between the basal 15, and 30 groups. In the case of CS, which has a high degree of postoperative pain, it is beneficial to control acute pain by properly setting the regimen of the initial IVA with a basal rate infusion to nullify a second prescription.

## 1. Introduction

Intravenous patient-controlled analgesia (IV PCA; IVA) using opioids is widely used for postoperative pain control.^[1]^ PCA refers to the preprogrammed doses of medications via an infusion pump that considers variables such as the initial loading dose, demand dose, lockout interval, and background infusion rate, and patients are instructed to press the demand button whenever necessary.^[2]^ The recommended demand and continuous doses were determined according to the type of opioid used in the IVA.^[1]^ Therefore, it is important to determine the appropriate variables for IVA, as well as the type of medications to be included, according to the expected severity of pain after surgery.

In 1 study, the pain scores for cesarean section (CS) were the ninth highest among 179 surgeries.^[3]^ Unproperly controlled postdelivery acute pain can lead to chronic pain and postpartum depression.^[4]^ At our hospital, we recognized that a more effective IVA regimen is needed during the hospitalization period following CS. As a second prescription, IVA is not economical, incurring the additional cost of infusion devices, and drugs. It is also not beneficial for pain control because of delays in patient administration. We assumed that the rate of a required second prescription would help predict adequate pain management. We propose that the IVA regimen can be considered suitable for acute pain control when the second prescription rate of IVA is maintained below 5% after CS without increasing significant adverse events.

This study investigated whether changes in the IVA regimen, such as opioid doses, addition of other analgesics, and pump mode, could reduce the need for a second prescription in patients with CS. We also investigated the effect of varying the dose of the basal infusion on the second prescription and side effects of IVA.

## 2. Methods

### 2.1. Patient population and postoperative analgesia

This study was approved by the Institutional Review Board of our hospital (Ref. SCMC 2022-07-004), and registered with the International Clinical Trials Registry Platform (http://cris.nih.go.kr). All patients scheduled for elective CS between January 2019 and August 2021 were reviewed. Patients were excluded from the study when their clinical information was insufficient or when the IVA device was broken. We subdivided patients according to the period when the regimen of IVA was changed as follows (see Supplemental Digital Content, http://links.lww.com/MD/I762, which records the regimen of IVA).

• Phase 1: Fentanyl bolus:12.00 to 52.80 mcg; basal:10.00 to 33.25 mcg; and total volume:60 to 200 mL
• Phase 2: Fentanyl bolus:26.60 to 46.80 mcg; basal:13.30 to 31.25 mcg, paracetamol 3 g, nefopam 60 mg; and total volume 150 to 170 mL

The IVA was a programmed bolus of 1 to 2 cc, continuous infusion of 1 to 2 cc, and lockout time of 8 to 10 minutes.

In addition, rescue analgesics such as meperidine and paracetamol were prescribed by the obstetrician when the patient complained of more than 5 points on the numerical rating scale for pain despite the administration of IVA in the ward.

### 2.2. Statistical analysis

Continuous variables are presented as median (25–75th percentiles) or means ± standard deviations. The 2 phases and groups were compared using the Mann–Whitney rank-sum test or independent *t* test. Categorical variables were compared between the 2 phases and groups using descriptive analysis and Fisher exact test, and are presented as percentages. All statistical analyses were performed using SigmaPlot 14.5 for Windows (Systat Software, Inc., Chicago, IL) and Stata 15.1 (Stata Corporation, College Station, TX). Statistical significance was set *P* value < .05.

## 3. Results

Overall, 632 patients were prescribed an IVA during the study period. The analysis was performed on all but 1 patient who was excluded because of malfunction of the IVA device.

Patient characteristics are summarized in Table 1. The average body weight in phase 2 was significantly higher than that in phase 1; however, there were no significant differences in the body mass index between the 2 phases.

**Table 1:** Patients characteristics.

	Phase 1 (n = 313)	Phase 2 (n = 318)	*P* value
Age (yr)	34.00 (31.00–36.00)	34.00 (31.00–36.25)	.901
Height (cm)	161.00 (158.00–164.00)	161.00 (158.00–165.00)	.161
Weight (kg)	70.00 (62.00–76.25)	71.00 (64.00–80.00)*	.019
Body mass index (kg/m^2^)	26.80 (24.30–29.70)	27.10 (25.00–30.00)	.116

* There is significant difference vs phase 1 (*P* < .05).

As shown in Table 2, the basal rate, bolus dose, total fentanyl dose, and total volume of IVA significantly increased in Phase 2. Consequently, the IVA infusion period was significantly extended. In terms of fentanyl dose in the second prescription IVA, there were no statistically significant differences between the phases, although the total dose was higher in phase 1 after the additional prescription.

**Table 2:** Comparison of changed variables, the rate of second prescription, discarded and side effects in intravenous patient-controlled analgesia (IVA) and rescue analgesics in the ward within postoperative 24 hours.

	Phase 1 (n = 313)	Phase 2 (n = 318)	*P* value
Basal rate (mcg/h)	15.00 (14.3–15.0)	31.25 (15.6–31.25)*	<.001
Bolus dose (mcg)	15.00 (15.0–26.6)	31.25 (31.20–31.25)*	<.001
Total fentanyl dose (mcg)	1500 (1500–2000)	2500 (2500–2500)*	<.001
Total volume (mL)	100 (90.0–150.0)	160 (160.0–160.0)*	<.001
Total infusion time (h)	35.00 (24.00–53.50)	60.00 (48.00–70.00)*	<.001
Total fentanyl dose including second prescription (mcg)	2000 (1500–2500)	2500 (2500–2500)*	<.001
IV PCA second prescription			<.001
Yes	86 (27.48)	12 (3.77)*	
No	227 (72.52)	306 (96.23)	
IV PCA drug discarded			.135
Yes	3 (0.96)	8 (2.52)	
No	310 (99.04)	310 (97.48)	
Side effects			<.001
Yes	3 (0.96)	22 (6.92)*	
Nausea	3	11	
Pruritis	0	4	
Dizziness	1	10	
No	310 (99.04)	296 (93.08)	
Additional pain control in ward within postoperative 24 h			<.001
Meperidine (25 mg)			
Yes	46 (14.70)	6 (1.89)*	
No	267 (85.30)	31 (98.11)	
Paracetamol (1000 mg)			
Yes	153 (48.88)	4 (1.26)*	
No	160 (51.12)	314 (98.74)	

* There is significant difference vs phase 1 (*P* < .05).

The second prescription rate of IVA in phase 2 was 3.77%, which was significantly lower than that in phase 1 (27.48%); however, the incidence of complications such as nausea, pruritus, and dizziness in phase 2 was 6.92%, which was significantly higher than that in phase 1 (0.96%) (Table 2). However, there were no significant differences between the 2 phases in the discontinuation of IVA (Table 2, *P* = .135).

The prescription rates of rescue analgesics meperidine (1.89%) and paracetamol (1.26%) administered in the wards within 24 hours of operation were significantly lower in phase 2 than in phase 1 (14.70% and 48.88%, respectively, in phase 1).

In phase 2 patients, the basal rate in the basal 30 group was almost double that in the basal 15 group, and there was also a significant difference in the bolus dose (Table 3). The total infusion time for IVA was 56 hours in the basal 30 group, which was significantly shorter than that in the basal 15 group (66 hours). However, there were no significant differences in the rate of a second prescription being required, drugs removed from the IVA regimen, or adverse events between the basal 15 and 30 groups (Table 3).

**Table 3:** Comparison of different basal rate infusions of IVA in phase 2.

	Basal 15 (n = 132)	Basal 30 (n = 186)	*P* value
Basal rate (mcg/h)	15.60 (15.60–15.60)	31.25 (31.25–31.25)*	<.001
Bolus dose (mcg)	31.20 (31.20–31.20)	31.25 (31.25–31.25)*	<.001
Total fentanyl dose (mcg)	2500 (2500–2500)	2500 (2500–2500)	1.000
Total volume (mL)	160 (160.00–160.00)	160 (160.00–160.00)	.233
Total infusion time (h)	66.00 (54.00–80.50)	56.00 (45.00–65.00)*	<.001
Total fentanyl dose including second prescription (mcg)	2500 (2500–2500)	2500 (2500–2500)	.117
IV PCA second prescription			
Yes	2 (1.52)	10 (5.38)	.075
No	130 (98.48)	176 (94.62)	
IV PCA drug discarded			
Yes	2 (1.52)	6 (3.23)	.477
No	130 (98.48)	180 (96.77)	
Side effects			.612
Yes	8 (6.06)	14 (7.53)	
No	124 (93.94)	172 (92.47)	

IVA = intravenous patient-controlled analgesia.

* There is significant difference vs Phase 1 (*P* < .05).

## 4. Discussion

When a combination of fentanyl 2500 mcg (total volume 160 mL), nefopam 60 mg, and acetaminophen 3 g in IVA for acute pain control after CS was prescribed with a basal rate of about 31.25 mcg/hours, bolus dose of about 31.25 mcg, and lockout time of 10 minutes, the second prescription rate for IVA was 1.52%. This was the result of satisfying the second IVA prescription rate set in this study, below 5%.

After a mother gives birth via CS, it is important to reduce pain to the extent that the mother can care for her baby. However, at the same time, it is also important to tailor the treatment plan so as to not affect breastfeeding and to reduce maternal complications caused by analgesics.^[5]^

The pain induced by CS consists of 2 types: somatic and visceral pain, known as incisional wound pain, and uterine contraction pain. Because these 2 pain mechanisms are different, the pain relief effects are also different, even with the same drug; therefore, it is better to use several drugs or methods that act on a specific pain mechanism.^[6,7]^ After CS, multimodal analgesic regimens that use opioids, nonsteroidal anti-inflammatory drugs, acetaminophen/paracetamol, ketamine, and gabapentin are preferred. Intraoperative interventions such as intrathecal and epidural opioids, regional nerve infiltration, and continuous wound infiltration with local anesthetics are also recommended.^[5]^ However, the method of pain management after CS may be influenced by the preference and proficiency of anesthesiologists and obstetricians, the availability of analgesics, and financial limitations.^[8]^ Therefore, it is important to consider these factors to establish the most suitable and efficient method for acute pain control after CS.

At our hospital, we primarily prescribed IVA for acute pain relief to all patients after CS. However, the regimen is not constant among anesthesiologists, and the increase in the second prescription of IVA suggests that the doses of analgesics and total volume of IVA were insufficient. Thus, we increased the total dose of fentanyl according to the total amount of fentanyl prescribed in the first and second IVA regimens before the study period and in phase 1, as well as the total volume and bolus dose of IVA. As a result, the total infusion time of IVA was extended significantly, and the rate of the second prescription decreased significantly in phase 2, indicating that this regimen can alleviate acute pain at least 48 hours after CS, after which analgesia can be converted into oral medications only.

At the same time, for phase 2 patients, the IVA regimen was supplemented with nefopam 60 mg and paracetamol 3 g in cases where the total infusion time would end within 24 hours. Multimodal analgesic approaches such as nefopam and/or paracetamol combined with opioid and non-opioid analgesics would enhance the morphine-sparing effect and pain relief, and would reduce opioid-related adverse events.^[9–12]^ However, Cuvillon et al^[13]^ suggested that nefopam in combination with paracetamol had limited opioid-sparing effects after open abdominal surgery. In this study, it would be difficult to conclude that nefopam and paracetamol facilitated acute pain control after CS, as these 2 drugs were added while increasing the total volume and opioid doses in the IVA, so their beneficial effects may have been occluded.

The basal rate of IVA was increased by some anesthesiologists in phase 2, and patients were classified into basal 15 and 30 groups in the analysis. Sinatra et al suggested that adding basal infusion to PCA was effective in managing postoperative pain, although mild side effects were more common. On the other hand, many studies conducted with morphine have demonstrated that adding constant infusion to IVA only increases drug dose and total opioid consumption, with no benefits in pain control.^[14–17]^ Moreover, the setting of basal infusion increases the incidence of respiratory depression as well as side effects, such as postoperative nausea and vomiting, dizziness, urinary retention, pruritus, and sedation.^[18,19]^ As a result, Notcutt et al^[18]^ implied that it would be effective to increase the bolus dose after surgery that causes pain requiring a high concentration of opioid, and it seems logical that the success of IVA is most affected by the demand dose.^[1]^ Fentanyl, a short-acting opioid, is suitable as a demand dose of IVA due to its fast onset; however, its offset is also fast, and the need for a basal dose has been suggested for appropriate pain relief.^[14]^ Recently, Hwang et al^[20]^ indicated that the application of an infusion device for fentanyl PCA programmed to automatically increase or decrease the baseline dose according to the patient’s needs is more effective in managing postoperative pain on an individual basis. However, similar to the results of morphine PCA studies, fentanyl PCA has also been found to increase fentanyl consumption and opioid-induced side effects when continuous infusion is initiated.^[21,22]^ Although the bolus dose used in our study differed significantly between the basal 15 and 30 groups, we decided to focus on the basal rate because we assumed that the bolus dose had little effect on the results. We assumed that the increase in the basal rate in the basal 30 group increased the total infusion time as well as the need for a second prescription of IVA; however, there were no statistically significant differences between the 2 groups. In addition, in the basal 30 group, the rate of side effects and discontinuation of IVA increased to 7.53% and 3.23%, respectively; however, this difference was not statistically significant between the 2 groups. Therefore, although a study with an additional pain evaluation scale is needed in the future, our results suggest that if severe pain is expected after surgery, such as CS, the appropriate basal infusion rate for fentanyl-based IVA must be carefully anticipated.

This study has several limitations. First, we did not find any documentation of visual analog scale values except for those included in the numerical rating scale of 5 or higher when the patient was prescribed rescue analgesics in the ward after CS. Second, the IVA regimen changes were not instigated sequentially; thus, the extent to which nefopam and paracetamol influenced the primary outcome of this study. Third, we did not subdivide and analyze the patients according to the anesthetic protocol, which could have affected the IVA dose. In future studies, it will be necessary to investigate the effect of multimodal analgesia on IVA dosages and to determine whether the IVA regimen should vary depending on the anesthetic protocols after CS.

In conclusion, we focused on the rate of the second prescription as an adjunct pain measure in IVA, which has not been well-described in previous studies. We considered the reduction of the second IVA prescription to indicate that an appropriate IVA regimen had been established. As a result, the proper IVA regimen, which minimized the second prescription for 48 hours after surgery, increased the total infusion time and reduced the prescription of rescue analgesics in the ward, which would be more effective in managing severe pain during the early postoperative period. In surgeries where opioid consumption is expected to be significant, such as in CS, it will be beneficial to control acute pain by properly setting the regimen of the initial IVA with a basal rate infusion that is sufficient to negate the second prescription.

## Author contribution

**Conceptualization:** Jeong Won Park, Jeong Yeon Kim.

**Data curation:** Sungbo Shim, Jihoon Lee.

**Formal analysis:** Jeong Won Park, Jeong Yeon Kim.

**Investigation:** Moon Ok Lee, Haeng Seon Shim.

**Supervision:** Mi Roung Jun, Haeng Seon Shim.

**Writing – original draft:** Mi Roung Jun.

**Writing – review & editing:** Mi Roung Jun, Moon Ok Lee.

## Supplementary Material



## References

[1] Palmer PP Miller RD. Current and developing methods of patient-controlled analgesia. Anesthesiol Clin. 2010;28:587–99. 21074739 10.1016/j.anclin.2010.08.010

[2] Momeni M Crucitti M De Kock M. Patient-controlled analgesia in the management of postoperative pain. Drugs. 2006;66:2321–37. 17181375 10.2165/00003495-200666180-00005

[3] Gerbershagen HJ Aduckathil S van Wijck AJ. Pain intensity on the first day after surgery: a prospective cohort study comparing 179 surgical procedures. Anesthesiology. 2013;118:934–44. 23392233 10.1097/ALN.0b013e31828866b3

[4] Eisenach JC Pan PH Smiley R. Severity of acute pain after childbirth, but not type of delivery, predicts persistent pain and postpartum depression. Pain. 2008;140:87–94. 18818022 10.1016/j.pain.2008.07.011 PMC2605246

[5] Kerai S Saxena KN Taneja B. Post-caesarean analgesia: what is new? Indian J Anaesth. 2017;61:200–14. 28405033 10.4103/ija.IJA_313_16 PMC5372400

[6] Hsu HW Cheng YJ Chen LK. Differential analgesic effect of tenoxicam on the wound pain and uterine cramping pain after cesarean section. Clin J Pain. 2003;19:55–8. 12514457 10.1097/00002508-200301000-00007

[7] Lavoie A Toledo P. Multimodal postcesarean delivery analgesia. Clin Perinatol. 2013;40:443–55. 23972750 10.1016/j.clp.2013.05.008

[8] McDonnell NJ Keating ML Muchatuta NA. Analgesia after caesarean delivery. Anaesth Intensive Care. 2009;37:539–51. 19681409 10.1177/0310057X0903700418

[9] Evans MS Lysakowski C Tramer MR. Nefopam for the prevention of postoperative pain: quantitative systematic review. Br J Anaesth. 2008;101:610–7. 18796441 10.1093/bja/aen267

[10] Girard P Chauvin M Verleye M. Nefopam analgesia and its role in multimodal analgesia: a review of preclinical and clinical studies. Clin Exp Pharmacol Physiol. 2016;43:3–12. 26475417 10.1111/1440-1681.12506

[11] Moon JY Choi SS Lee SY. The effect of nefopam on postoperative fentanyl consumption: a randomized, double-blind study. Korean J Pain. 2016;29:110–8. 27103966 10.3344/kjp.2016.29.2.110 PMC4837116

[12] Freo U. Paracetamol for multimodal analgesia. Pain Manag. 2022;12:737–50. 35440176 10.2217/pmt-2021-0116

[13] Cuvillon P Zoric L Demattei C. Opioid-sparing effect of nefopam in combination with paracetamol after major abdominal surgery: a randomized double-blind study. Minerva Anestesiol. 2017;83:914–20. 28192892 10.23736/S0375-9393.17.11508-7

[14] Owen H Szekely SM Plummer JL. Variables of patient-controlled analgesia. 2. Concurrent infusion. Anaesthesia. 1989;44:11–3. 2929900 10.1111/j.1365-2044.1989.tb11088.x

[15] Parker RK Holtmann B White PF. Effects of a nighttime opioid infusion with PCA therapy on patient comfort and analgesic requirements after abdominal hysterectomy. Anesthesiology. 1992;76:362–7. 1539846 10.1097/00000542-199203000-00007

[16] Parker RK Holtmann B White PF. Patient-controlled analgesia. Does a concurrent opioid infusion improve pain management after surgery? JAMA. 1991;266:1947–52. 1895471 10.1001/jama.266.14.1947

[17] Dal D Kanbak M Caglar M. A background infusion of morphine does not enhance postoperative analgesia after cardiac surgery. Can J Anaesth. 2003;50:476–9. 12734156 10.1007/BF03021059

[18] Notcutt WG Morgan RJ. Introducing patient-controlled analgesia for postoperative pain control into a district general hospital. Anaesthesia. 1990;45:401–6. 2356938 10.1111/j.1365-2044.1990.tb14787.x

[19] Schug SA Torrie JJ. Safety assessment of postoperative pain management by an acute pain service. Pain. 1993;55:387–91. 8121701 10.1016/0304-3959(93)90016-I

[20] Hwang J Min SK Chae YJ. Continuous fentanyl background infusion regimen optimised by patient-controlled analgesia for acute postoperative pain management: a randomised controlled trial. J Clin Med. 2020;9:211. 31941001 10.3390/jcm9010211 PMC7019224

[21] Kim K. Analysis of the current state of postoperative patient-controlled analgesia in Korea. Anesth Pain Med. 2016;11:28–35.

[22] Jung H Lee KH Jeong Y. Effect of fentanyl-based intravenous patient-controlled analgesia with and without basal infusion on postoperative opioid consumption and opioid-related side effects: a retrospective cohort study. J Pain Res. 2020;13:3095–106. 33262644 10.2147/JPR.S281041 PMC7699445

